# A Role for Estrogen in Schizophrenia: Clinical and Preclinical Findings

**DOI:** 10.1155/2015/615356

**Published:** 2015-09-27

**Authors:** Andrea Gogos, Alyssa M. Sbisa, Jeehae Sun, Andrew Gibbons, Madhara Udawela, Brian Dean

**Affiliations:** ^1^Florey Institute of Neuroscience and Mental Health, University of Melbourne, Parkville, VIC 3010, Australia; ^2^School of Psychology and Public Health, La Trobe University, Bundoora, VIC 3086, Australia

## Abstract

Gender differences in schizophrenia have been extensively researched and it is being increasingly accepted that gonadal steroids are strongly attributed to this phenomenon. Of the various hormones implicated, the estrogen hypothesis has been the most widely researched one and it postulates that estrogen exerts a protective effect by buffering females against the development and severity of the illness. In this review, we comprehensively analyse studies that have investigated the effects of estrogen, in particular 17*β*-estradiol, in clinical, animal, and molecular research with relevance to schizophrenia. Specifically, we discuss the current evidence on estrogen dysfunction in schizophrenia patients and review the clinical findings on the use of estradiol as an adjunctive treatment in schizophrenia patients. Preclinical research that has used animal models and molecular probes to investigate estradiol's underlying protective mechanisms is also substantially discussed, with particular focus on estradiol's impact on the major neurotransmitter systems implicated in schizophrenia, namely, the dopamine, serotonin, and glutamate systems.

## 1. Introduction

Schizophrenia is a complex neuropsychiatric disorder, which will affect approximately 0.7% of the population during their lifetime [1]. Schizophrenia is a profoundly debilitating illness currently ranking among the top 10 causes of long-term disability worldwide [2], which imposes a significant financial burden on public health services as it is one of the most financially costly psychiatric disorders to manage [3]. The profound impact of the disorder is reflected in the fact that approximately 70–92% of patients with schizophrenia are unemployed [4]. Additionally, people with schizophrenia are 13-fold more likely to die by suicide than are members of the general population [5], and also have a life expectancy 10–25 years less, even after accounting for suicide [6]. Schizophrenia is a disorder characterised by severe impairment of cognition, emotions, and behaviour [2] with symptomatology being classified within three main clusters: positive symptoms, negative symptoms, and cognitive deficits [7]. Positive symptoms (additive to normal, healthy function) include hallucinations and delusions whilst negative symptoms (deficits) include blunted drive and affect (i.e., asociality, alogia, and avolition) [8]. The heterogeneity of symptoms reflects the diagnosis of schizophrenia defined as a syndrome of disorders, thereby signifying that there may be a need to identify differing aetiologies for different diseases within the syndrome of schizophrenia, which currently remain largely unknown [9].

The numerous neurotransmitter systems implicated in schizophrenia (e.g., dopaminergic, glutamatergic, and serotonergic systems) add to the difficulty in effectively treating the disorder [10]. Presently, the primary treatment for schizophrenia is antipsychotic medications [11], which predominantly target the dopaminergic system [12]. However, antipsychotics have reduced efficacy on the negative and cognitive symptoms of schizophrenia [11] and with respect to the positive symptoms are not efficacious for 20–30% of patients with the disorder [10, 13]. This has led to the exploration of potential adjunctive treatments, such as treatment with estrogen [14], to extend the current therapeutic benefits of antipsychotic drugs. Importantly the gender differences in onset, symptom severity, and outcome of schizophrenia are now thought to support the hypothesis that sex hormones may also have a role in the aetiology, as well as treatment, of schizophrenia.

Estrogen is a gonadal hormone that can exert powerful effects in numerous regions of the brain, consequently affecting mood, cognition, and behaviour [15]. Research over the last two decades has established a clear neuromodulatory role of estrogen in the pathogenesis and therapeutics of neuropsychiatric disorders including schizophrenia. Estrogen is often considered the primary “female” sex hormone, although it is present in both sexes [16]. Reference to estrogen can broadly refer to numerous estrogenic compounds including estradiol, estrone, estriol, equilin, and ethinylestradiol. In this review, reference to estrogen refers to the most potent endogenous form, 17*β*-estradiol, unless otherwise stated. 17*β*-estradiol has a role in the development of secondary sex characteristics in women and reproduction in men and in both sexes has peripheral effects in areas including the liver and bone [17]. While 17*β*-estradiol is primarily produced in the ovaries to regulate menstrual cycle in females, it is also created by nonendocrine tissues, including fat, breast, and importantly the brain [18]. Estrogen has neuroprotective properties and it has been suggested that it can exert its effects over the entire lifetime, protecting the brain from certain insults [19]. Accumulating evidence has led to the hypothesis that recurring hormone influxes in women serve as a protective factor in the initial development of schizophrenia [14]. Thus, in recent years an increasing amount of literature has explored estrogen therapy as a potential form of treatment for schizophrenia [20, 21].

This literature review aims to critically analyse the relevance of estrogen in relation to the pathogenesis and therapeutics of schizophrenia in a clinical setting. This review will also report on preclinical research and the molecular mechanisms that may underlie the therapeutic effects of estrogen in schizophrenia.

## 2. Clinical Findings

### 2.1. Gender Differences in Schizophrenia

A rich literature elaborately describes gender differences in schizophrenia relating to disease risk, course, and outcome [22–24]. There is a difference in the age-at-onset of schizophrenia between the sexes, whereby men reach a peak onset at the ages of 18–24 years, whereas for women it occurs up to 4 years later [25, 26]. This is a well-replicated finding and occurs regardless of the definition of onset used; it is consistent across cultures and is not due to differences in symptoms or social role. Furthermore, only in females with schizophrenia is there a second peak age-at-onset at 45–50 years of age [27]. There is an increased incidence rate in men (1.4 : 1 ratio), which has been verified by 2 independent meta-analyses, and remains even after controlling for various confounding factors such as age range, diagnostic criterion, and hospital bias [28, 29]. There is a plethora of studies based in different countries and cultures supporting the notion that women with schizophrenia present with a less severe course of the illness compared to men [24, 30, 31]. For example, women with schizophrenia present with less severe negative symptoms but exhibit more positive and affective symptoms [32]. In women, a later age-at-onset and presentation of affective symptoms have predicted a better prognosis, whereas in men an earlier onset and presentation of primarily negative symptoms predict a worse course of illness and outcome [22]. Females show a more favourable antipsychotic treatment response than males [33, 34], have fewer hospitalisations, better adapt to the illness, and present less disability (particularly with self-care). Women also have an improved outcome and improved quality of life; for example, they are more likely to be married, remain employed, and keep in contact with family and friends [24]. Men have more brain structural abnormalities than females, including enlarged ventricles and decreased temporal lobe volume [35–37].

### 2.2. Estrogen Hypothesis of Schizophrenia

In light of the gender differences described above, it has been hypothesized that gonadal steroids may play a role in buffering females against the development of schizophrenia [32, 38]. This is consistent with the existence of a second peak of onset in females after the age of 40 which may be associated with menopause, a time of rapidly declining sex hormone levels [25, 39]. Further, premenopausal women with schizophrenia experience a better course of illness with less negative symptoms and respond better to antipsychotic treatment (i.e., require lower doses) than older women [34]. The most common interpretation of these gender differences is the well-described “estrogen hypothesis,” which postulates that estrogen plays a protective role against schizophrenia [14, 40]. However, it is important to note that the studies describing gender differences in schizophrenia suggest sex steroid dysfunction, not necessarily only estrogen dysfunction. A number of reproductive hormones may be implicated, including testosterone, progesterone, or luteinising hormone, and thus it is important to acknowledge that there is a complex interplay of hormones occurring. For example, progesterone and estrogen naturally vary with each other over endogenous hormonal cycles; therefore the influence of progesterone or an interaction between the two hormones on the observed phenomena cannot be excluded. However, this review will focus on estrogen as the estrogen hypothesis has been well-supported by molecular, animal, and clinical studies [41–43].

### 2.3. Evidence for Estrogen Dysfunction in Patients with Schizophrenia

An early study reported that, of the sample of 276 women admitted to psychiatric hospitals, 46% were admitted during or immediately before menstruation, a period of low circulating estrogen levels [44]. Further, psychotic symptoms were reported to improve during pregnancy [45] but worsened postpartum [46]. More recently, case reports and clinical studies have shown that women with schizophrenia demonstrate increased symptom severity, greater relapse rates, and more hospital admissions during times of low circulating sex hormones, including the early follicular phase of the menstrual cycle, postpartum, and postmenopause [47–50]. In contrast, rates of relapse are less frequent and symptom severity is reduced during times of high circulating sex hormones, including pregnancy and the mid-luteal stage of the menstrual cycle [47, 51]. For example, Hallonquist et al. [52] assessed the variation in symptom severity in female outpatients with schizophrenia during two phases of the menstrual cycle. The authors found that symptom scores as measured by the Abbreviated Symptom Checklist were distinctly low during the mid-luteal phase but high during the early follicular phase [52]. Similarly, Rubin et al. [50] reported that female patients with chronic schizophrenia showed less severe positive symptoms and general psychopathology (measured using the Positive and Negative Syndrome Scale, PANSS) during the mid-luteal phase versus the early follicular phase, whereas negative symptom severity did not change across the cycle [50]. Some studies have specifically shown that there is a negative correlation between circulating estrogen levels and symptoms of schizophrenia, particularly the positive symptoms [39, 53]. In 125 premenopausal women with schizophrenia, Bergemann and colleagues [53] assessed psychopathology scores three times during the menstrual cycle. Using the PANSS and Brief Psychiatric Rating Scale, they found a significant improvement in psychotic symptoms during the luteal phase, which was associated with estradiol plasma levels [53]. A positive relationship has also been discovered between later menarche, higher negative symptom scores (as measured by the Scales for Assessment of Negative Symptoms), and greater functional impairment in women with schizophrenia, suggesting that earlier puberty might predict improved clinical outcome [54]. Similarly, a negative association has been discovered between age at menarche and first psychotic symptoms and first hospitalisation; more specifically, a relationship has been identified between earlier puberty and later onset of illness in women with schizophrenia [38].

Women with schizophrenia are often hypoestrogenic; that is, their circulating levels of estrogen are much lower than the normal reference range and they tend to experience menstrual irregularities [48, 49, 55]. Importantly, some studies showing reduced estrogen levels in women with schizophrenia were conducted during the preantipsychotic era [56, 57]. Since the introduction of antipsychotic drug treatment, reduction in estrogen levels is correlated with an increased risk of symptoms and is found regardless of the type of antipsychotic treatment [47, 58]. This is important as some antipsychotics can cause hyperprolactinaemia, which leads to a reduction in estrogen levels [59]. Hyperprolactinaemia is mainly associated with antipsychotics, such as risperidone, which predominantly block the dopamine D2 receptor, the receptor that modulates prolactin release from the pituitary [60].

An important question is whether estrogen dysfunction occurs prior to or after the onset of schizophrenia. Early puberty has been associated with a late onset of the disorder [38], suggesting that physiological estrogens might delay the onset of schizophrenia [56]. Many clinical studies examining plasma estrogen levels and symptomatology in schizophrenia patients require their participants to have a history of regular menstrual cycles; therefore it cannot be inferred from these samples whether gonadal dysfunction is merely a state or a trait of the disorder. Schepp [61] attempted to explore this question by investigating premenopausal first-episode schizophrenia patients. In comparison to age-matched healthy controls, schizophrenia patients had later menarche, mid-cycle bleeding, mild bleeding, hirsutism, and more tendency to be infertile [56, 61]. This study demonstrates evident gonadal dysfunction in a sample of first-time admitted patients; however, a longitudinal experiment examining endocrinological function, inclusive of prepubescent participants, is necessary to sufficiently answer whether premorbid hypoestrogenism occurs.

### 2.4. Clinical Trials of Adjunctive Estrogen in Schizophrenia

A growing body of double-blind, placebo-controlled, randomized trials provides evidence that estrogen treatment administered in conjunction with antipsychotics is beneficial for schizophrenia, particularly in reducing the positive symptoms [20, 62, 63]. An initial pilot study by Kulkarni et al. [64] discovered that the synthetic 17*β*-estradiol derivative, ethinylestradiol, taken orally daily for eight weeks significantly improved positive symptoms in premenopausal women with schizophrenia. Later trialling a transdermal method of administration, Kulkarni et al. [20, 63] found that women with schizophrenia receiving adjunctive estradiol had significant improvements in the positive symptoms and general psychopathology (PANSS) ratings [20, 63]. In their largest study to date, Kulkarni and colleagues [20] tested 183 premenopausal women with schizophrenia who were receiving transdermal 17*β*-estradiol (100 *μ*g/day or 200 *μ*g/day for 8 weeks) together with their prescribed antipsychotic. The largest effect was found in the women receiving 200 *μ*g of estradiol, who showed reduced scores on the positive subscale of the PANSS [20]. Another group found similar beneficial effects, where 8 weeks of adjunctive haloperidol and ethinylestradiol treatment resulted in reduced positive, general, and total PANSS scores, compared to the haloperidol-only group [62]. On the other hand, a study by Bergemann and colleagues [65] failed to replicate the beneficial effect of estradiol in their placebo-controlled, double-blind study with 46 hypoestrogenic women, finding there was no difference in PANSS scores, relapse rates, or antipsychotic dose between treatment and placebo. This may be due to the use of a combined 17*β*-estradiol (1–4 mg) and progestin treatment, with doses varying dependent on the phase of menstrual cycle [65]. In comparison, Kulkarni et al. [20, 63] administered only estrogen treatment and at a consistent daily dose for the duration of the experiment.

One cross-sectional study compared postmenopausal women with schizophrenia who were either users or nonusers of hormone replacement therapy. They found that the women taking hormones required a lower dose of antipsychotics and had less severe negative symptoms [66]. Research thus far has primarily concerned females, evidently due to the premise for estrogen therapy relying on observation of hypoestrogenism in women. One study that examined the effects of estradiol in men with schizophrenia found that after two weeks of oral estradiol treatment in conjunction with antipsychotics, the estrogen group experienced more rapid reduction in general psychopathology compared to the placebo group [67]. Although there is concern regarding the potentially feminising side effects of estradiol, estrogen therapy is currently used in males for other clinical conditions (e.g., prostate cancer), and the results of the Kulkarni et al. [67] study suggest exploration of estrogen treatment in men with schizophrenia is warranted.

Other forms of estrogen have also shown some beneficial effects on schizophrenia symptoms, although perhaps not as potent as the effects of 17*β*-estradiol. In a double-blind, randomized, placebo-controlled trial, 32 premenopausal women with chronic schizophrenia were treated with conjugated estrogens for 4 weeks, in addition to their antipsychotic treatment. Participants experienced a significant decrease in positive, negative, general, and total PANSS scores [68]. In a similar study, however, Louzã et al. [69] found no significant difference between the treatment and placebo groups, although there was a trend for the conjugated estrogen group to show greater improvement [69]. The selective estrogen receptor modulator raloxifene has also been trialled in women with schizophrenia with favourable results for the positive [70, 71], negative [72], and cognitive symptoms [73, 74].

Clinical research specifically concerning the influence of estradiol on cognition in schizophrenia patients is limited. With relevance to endogenous estrogen, research has found estradiol can improve certain cognitive functions in women with schizophrenia. Hoff et al. [75] determined there was a positive correlation with serum estradiol levels and a global cognitive score including six cognitive domains, with verbal and spatial memory, and perceptual motor speed being the most strongly related. Ko and colleagues [76] stratified their sample of women with schizophrenia into low or high estrogen groups by using the normal serum reference ranges for estradiol during the follicular phase of the menstrual cycle. Similar to the results of Hoff et al. [75], they found diminished performance in verbal memory and executive function in the low estradiol group, compared to the high estradiol group. Studies administering estradiol treatment provide less consistent results. Bergemann and colleagues [77] found oral 17*β*-estradiol and adjunctive antipsychotic treatment for women with schizophrenia improved metaphoric speech but had no effect on word fluency and verbal ability. In an experiment examining transdermal 17*β*-estradiol, Kulkarni et al. [20] found there were no significant differences between or within groups in cognitive domains including attention, language, visuospatial/constructional ability, and memory. These studies employed different neurocognitive batteries and different methods of administering estradiol, which may account for dissimilar outcomes.

The diverse results in the aforementioned estradiol trials may be due to a variety of inconsistent factors including dissimilar measures, severity of symptoms, variable treatment duration, additional pharmacotherapy (i.e., antipsychotics, benzodiazepines), oral versus transdermal administration, and pharmacological and pharmacokinetic variation in estrogen. Additionally, despite its putative effect on the positive symptoms of schizophrenia, estradiol at the efficacious dose is unfortunately not feasible for long-term management of schizophrenia due to the associated health risks (e.g., thromboembolism, endometrial hyperplasia). Evidently, estradiol treatment in men with schizophrenia also remains controversial due to potential feminising side effects [20, 67]. Nevertheless, overall the epidemiological and clinical data presented provide strong support for the notion that estradiol is protective in women with schizophrenia, particularly for the positive symptoms.

## 3. Mechanisms of Estrogen Action in Schizophrenia

**Figure 1:**
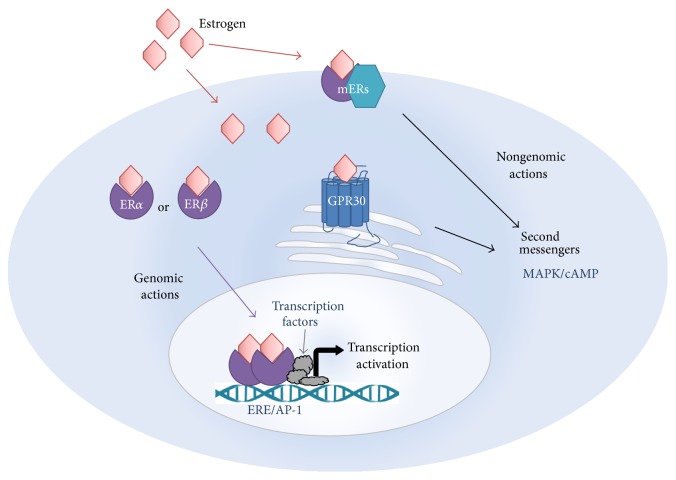
Putative mechanisms of estrogen action in the cell. Estrogen can act via either genomic or nongenomic mechanisms. Genomic mechanisms involve activation of the estrogen receptors (ERs) by estrogen, which then translocate to the cell nucleus as hetero- or homodimers to bind to estrogen response elements (EREs) or to activator protein 1 (AP-1) sites, resulting in transcription activation. Nongenomic actions occur via binding of estrogen to ERs or to a G protein coupled receptor GPR30, either intracellularly or at the plasma membrane (mERs) to activate second messenger systems, such as those involving mitogen-activated protein kinase (MAPK) or cyclic adenosine 3′,5′-monophosphate (cAMP) pathways, which can also activate transcription or have other effects.

The molecular mechanisms of how estrogen may affect schizophrenia symptoms remain largely unknown. Perhaps the simplest explanation is that estrogen can regulate the dopaminergic system of the central nervous system (CNS) by affecting the expression and function of dopamine receptors and transporters [78, 79]. However, there are several other possible mechanisms by which estradiol can exert the effects in the CNS, some of which have been well defined and others are yet to be characterised. Estradiol actions are generally categorised as either genomic or nongenomic. Genomic actions are delayed in onset and prolonged in duration, such as those likely to occur after chronic estradiol treatment. These effects occur through binding of intracellular estradiol to the estrogen receptor (ER), which belongs to the nuclear receptor superfamily. Upon binding, the receptors dimerise and then translocate to the nucleus where they bind to specific DNA sequences called estrogen response elements (EREs) found in the promotor region of estrogen-responsive genes or to activator protein 1 (AP-1) sites via Fos/Jun interactions, resulting in transcriptional activation of many different genes. The nongenomic actions occur through activation of intracellular second messenger pathways, such as the MAP kinase and cAMP, to elicit a more rapid response, including cell-excitability, synaptic transmission, and antioxidant effects [80–82]. These are believed to be mediated via either ERs interacting with other proteins to form a large complex anchored to the plasma membrane or an alternative G protein coupled receptor, GPR30 [83, 84] (see Figure 1).

Gene profiling of the mouse brain after treatment with estradiol has revealed changes in genes associated with biosynthesis, growth, synaptic potentiation and myelination, lipid synthesis and metabolism, cell signalling pathways, and epigenetic modifications [85, 86]. In the primate prefrontal cortex, estrogen treatment caused changes in genes involved in transcription regulation, neurotransmission, cell signalling, cell cycle control, and proliferation and differentiation [87]. These effects could be a result of either genomic or nongenomic actions [88]. Thus estrogen can have far-reaching and diverse effects on the brain. Interestingly, one group studied the gene expression profile of a cell line treated with 18 different antipsychotics and found a common signature shared by antipsychotics and estrogen receptor modulators: lipid homeostasis [89]. It is theorised that the estrogen pathway may be involved in the therapeutic effect of antipsychotics [89].

### 3.1. Estrogen Receptors

The first ER, ER*α*, was cloned in 1986 [90], and the second subtype, ER*β*, was not discovered until 1996 [91]. The two receptor subtypes are encoded by separate genes,* ESR1* and* ESR2, *respectively. A splice variant of ER*β* was later identified, ER*β*2 [92], which shows a much lower affinity to 17*β*-estradiol than ER*α* and ER*β*1 [93], but can competitively bind at EREs and as such can act as a negative regulator of estrogen-dependent transcriptional activation [94]. As expected, ER*α* is highly expressed in areas of the CNS that are implicated in the control of reproductive functions, such as the hypothalamus and preoptic areas [84], and receptor levels tend to be higher in the female rats than in males [95–97]. ER*β* expression shows much overlap with ER*α* but appears to be more widely distributed, showing strong expression in areas such as the hippocampus in mice, rats, and humans [91, 98], while ER*α* mRNA is abundantly found in the prefrontal cortex of nonhuman primates [87, 99, 100], indicating both these receptors are involved in potentiating the nonreproductive estrogen actions such as learning and memory.

For many years there was uncertainty surrounding the role of ERs in nongenomic actions of estrogen. While ER*α* and ER*β* were shown to activate nongenomic signalling through crosstalk with other signal transduction proteins [84], these receptors could not fully account for observed downstream effects of estrogen, such as its antioxidant properties [101], and it was speculated that another receptor could be involved. GPR30, previously an orphan receptor, was recently renamed G protein coupled estrogen receptor 1 (GPER) following evidence that estrogen can bind to and activate the receptor [102, 103]. It is found to be expressed in multiple regions of the rat CNS [104], including the hippocampus, frontal cortex, and substantia nigra [94, 104]. Several studies indicate that this receptor is localised to the cytoplasm [104], specifically the endoplasmic reticulum and Golgi apparatus [90, 105, 106]. However there is also evidence that it is expressed at the plasma membrane and dendritic spines of rat hippocampal neurons [104, 107–109], suggesting that localisation may be cell type-specific or influenced by state. This receptor can rapidly activate multiple kinase pathways involved in nongenomic estrogen actions [110] and appears to mediate many of the effects of estrogen in neuronal cells [111], including calcium oscillations and luteinising hormone-releasing activity in primate neurons [112]. While CNS expression patterns do not appear to differ between the adult male and female rat [104, 105, 113], the receptor is implicated in sexual dimorphism of immune response in the GPER knock-out mouse [114]. Importantly, there is emerging evidence for GPERs role in learning and memory [115–117] as well as neuronal plasticity [118]. Several new members and isoforms of estrogen receptors have also recently been identified, some of which are expressed in the CNS, such as ER-X, which could be involved in the nongenomic actions of estrogen [119]; however these have yet to be well characterised [120].

### 3.2. Estrogen Receptors and Cognition

The role of estrogen on cognition is of particular importance for schizophrenia as the cognitive deficits associated with the disease are considered the most debilitating symptoms for patients to assimilate into society [121], and these symptoms are poorly treated using current antipsychotics [122]. Sinopoli and colleagues [123] showed that a low dose of estradiol injected directly into the hippocampus, or a high dose injected into the prefrontal cortex, could improve radial arm maze performance (spatial working memory task) in rats [123]. Several studies have demonstrated contradicting findings on the roles of ER*α* and ER*β* on cognition. Viral delivery of ER*α* into the hippocampus has been shown to rescue memory deficits in ER*α* knock-out mice [124]. In contrast, another study has shown a negative effect of ER*α* on memory, where estradiol treatment impaired acquisition of spatial memory, but not cue discrimination, in the Morris water maze in ovariectomised wild-type mice but not ER*α* knock-out mice [123]. This suggests an ER*α*-dependent mechanism of estradiol in impairing spatial task performance [125]. Recently, mice with ER*β* knock-down showed improved spatial learning, which could be reversed by viral delivery of ER*β* to the hippocampus [126]. By contrast, treatment with selective ER*β* agonist, diarylpropionitrile (DPN), in female ovariectomised rats enhanced recognition memory but had no effect following treatment with ER*α*-selective agonist, propyl pyrazole triol (PPT) [127]. Results suggest that ER*β* mediates the subchronic and acute effects of estrogen on recognition memory. Molecular work by the same authors showed that memory enhancements via DPN are likely to occur through alterations in monoamines in the hippocampus and prefrontal cortex [127]. In a different species, Phan et al. [128] found that acute PPT treatment enhanced object and place recognition in ovariectomised mice, whilst DPN at the same dose did not affect object recognition and only slightly facilitated place recognition [128]. Collectively, research demonstrates that different estrogen agonists, estrogen receptors, and brain regions have the ability to mediate dissimilar forms of learning and memory. It is currently difficult to isolate specific actions of ERs in relation to cognitive function. Not only does function change dependent on the cognitive task, but factors such as age, sex, and treatment duration can also alter outcomes. The latter is of particular importance due to the influence of treatment period on genomic versus nongenomic outcomes and consequently mediation via different estrogen receptors [129]. More preclinical research is necessary to further clarify the specific role of ERs in relation to cognition, especially with specific relevance to schizophrenia.

### 3.3. Estrogen Effects on Brain Structure

Schizophrenia is associated with various structural brain changes, such as progressive decline in global gray and white matter volume in multiple brain regions followed by continuous ventricular enlargement [130]. Abnormal cytoarchitecture also commonly occurs, including neuronal soma and neuropil volume reductions, irregular synaptic organization, and ectopic neurons [131, 132]. The effects of estrogen treatment on brain structure have been well documented, including the modulation of neurogenesis, synaptic density, plasticity and connectivity, and axonal sprouting (reviewed in [84]). Of particular relevance to cognition, estradiol treatment has been shown to enhance hippocampal synaptic plasticity in young ovariectomised rats [133], induce dendritic spine formation in CA1 pyramidal neurons [134], and stimulate neurogenesis of granule cells in the dentate gyrus of adult female rats [135]. Estrogen can also modulate neurotrophic factors [136] as well as neurotransmission [15, 137], which can secondarily promote neuronal survival and proliferation. Thus, in women with schizophrenia, lower circulating estradiol levels [51, 65] may contribute to the observed brain pathology associated with the disorder. Based on these findings we would expect to see sex differences in brain abnormalities in people with schizophrenia. Indeed, two MRI studies reported more severe abnormalities in males than in females with the disorder when compared to age- and sex-matched controls, particularly in regard to ventricular enlargement [37] and temporal lobe volume [35]. However there are some conflicting reports, where a similar MRI study showed no difference [138]. Overall these studies suggest that estrogen levels could influence the brain structure differences that occur in the CNS of people with schizophrenia.

### 3.4. Neuroprotection by Estrogen

Neuroprotective effects are another key component of estrogen action that is relevant to schizophrenia [84]. Early cell culture studies showed increased neuronal survival upon treatment with estrogen under serum deprivation [139–142] and subsequent studies have shown estrogen protection against injury from excitotoxicity [143–145], oxidative stress [101, 146], inflammation [147, 148], and apoptosis [149]. Some of these protective actions have been attributed to the ability of estrogen to reduce the generation of free radicals [150]. More recently, it has been suggested that the neuroprotective actions of estrogen are mediated through maintaining mitochondria function [151], and there is growing evidence of mitochondrial dysfunction playing a role in schizophrenia [152]. Taken together, these findings indicate that low estrogen levels may leave the brain vulnerable to insult or age-related changes, leading to development of schizophrenia or increased symptom severity, and could explain the observed differences in disease onset and severity between males and females. Treatment with estrogen may therefore help to protect the brain from disease progression.

### 3.5. Changes in Estrogen Signaling in Schizophrenia

In 2005, Perlman and colleagues showed that while ER*α* mRNA was not different in the dorsolateral prefrontal cortex of people with schizophrenia, in the dentate gyrus region of the hippocampus ER*α* expression levels were lower in schizophrenia compared to healthy controls [153]. This has implications in estrogen driven synaptic plasticity and neurogenesis, as this region of the hippocampus is important for control of these activities. Further, lower receptor levels are unlikely to be the result of lower circulating estradiol levels as low levels of hormone would be expected to upregulate receptor expression [154]. Perlman et al. [153] study also detected a negative correlation between ER*α* mRNA expression in the dentate gyrus and age-at-onset, suggesting ER*α* levels may confer vulnerability to the disease. Furthermore, their finding appeared to be diagnosis specific, as people with major depressive disorder and bipolar disorder showed no difference in ER*α* expression compared to control [153]. On the other hand, ER*α* mRNA levels were lower in the amygdala in major depressive disorder and bipolar disorder, but not in schizophrenia, compared to control, while sex differences were detected in the dorsolateral prefrontal cortex of people with major depressive disorder that was not present in control. These findings illustrate that alterations in ER*α* expression in the CNS across major mental illnesses are specific to sex, region, and diagnosis [153]. More recently, ER gene variation has also been implicated in schizophrenia risk. Weickert et al. [155] showed a SNP in intron 1 of the* ESR1* gene was more prevalent in people with schizophrenia. Moreover, this SNP was associated with lower expression levels of ER*α* in the prefrontal cortex among people with the disorder [155]. Thus, ER*α* expression levels in the CNS appear to play an important role in schizophrenia pathophysiology and may explain some of the cognitive deficits associated with disorder, particularly in those CNS regions that are implicated in cognition such as the hippocampus [156] and prefrontal cortex [157].

### 3.6. Estrogen Effects on Major Neurotransmitter Systems Targeted by Antipsychotics

Several converging lines of evidence from clinical and animal studies suggest that estrogen can act to modulate the activity of the neurotransmitter systems targeted by current antipsychotics [30, 79, 83, 158]. Understanding the nature of these interactions is important for addressing the therapeutic potential of estrogen and of compounds that target estrogen signalling. Researchers have labelled estradiol as neuroprotective and antipsychotic, implicating numerous neurotransmitter systems in this mechanism [30]. The strongest evidence for estrogen modulation of neurotransmitter systems comes from studies examining the dopamine, serotonin, and glutamate systems; examples of these studies are described below.

#### 3.6.1. Estrogen Interaction with Dopamine

As stated earlier the most direct route by which estrogen could influence symptom severity in schizophrenia could be by modulating dopaminergic activity in the CNS as hyperactivation of the dopamine signalling system is thought to be a central mechanism affected in schizophrenia [12, 159]. Central to this hypothesis are observations that typical antipsychotics, such as haloperidol, are potent antagonists of dopamine D2 receptors [160] and can reduce positive symptoms of schizophrenia [161]. The stimulatory effect of estrogen on the activity of dopaminergic neurons, particularly those in the striatum and nucleus accumbens, is well documented (see [79]). Rodent studies have demonstrated that phases of dopaminergic transmission vary during the estrous cycle [162]. Removal of the primary source of estradiol via ovariectomy evokes a permanent loss of dopamine neuron density in the substantia nigra in nonhuman primates [163]. Estradiol treatment can modulate the levels of dopamine transporters and receptors (pre- and postsynaptic) and dopamine synthesis, release, and turnover in both cortical and striatal regions [162, 164–167]. For example, ovariectomy in rats has been shown to reduce protein levels of the dopamine active transporter (which reuptakes dopamine into the neuron for recycling or degradation) and increase levels of dopamine D2 receptor in the nucleus accumbens and caudate nucleus [78]. Subsequent treatment in ovariectomised rats with 17*β*-estradiol reversed the loss of dopamine transporter levels and reduced dopamine D2 receptor levels below that of intact control levels [78]. Chronic 17*β*-estradiol treatment of ovariectomised monkeys increased dopamine transporter expression levels in the caudate putamen compared to vehicle-treated monkeys [168] and also led to a downstream activation of the Akt/GSK3 signalling pathway, which is thought to be impaired in schizophrenia [169]. Ovariectomised macaques showed increased numbers of neurons expressing dopamine *β* hydroxylase (DBH), an enzyme involved in metabolising dopamine, across all layers of the dorsolateral prefrontal cortex. Treating ovariectomised macaques with 17*β*-estradiol returned the number of DBH-immunoreactive neurons to levels comparable to intact animals. Furthermore, cotreating ovariectomised macaques with 17*β*-estradiol and progesterone did not produce a greater effect than 17*β*-estradiol alone, suggesting this effect is primarily mediated by estrogen [170]. One study treated ovariectomised rats with an ER*β*-selective agonist and found increased brain monoamine levels in the prefrontal cortex, including a marked increase in dopamine levels, its metabolite homovanillic acid (HVA), and HVA/dopamine ratio [127]. In humans, a PET study did not show any significant variation in striatal D2 receptor density throughout the menstrual cycle [171]. However, postmenopausal women receiving estrogen replacement therapy following hysterectomy or oophorectomy showed increased dopamine responsiveness to apomorphine [172].

Rodent behaviour studies also show marked protective effects of estrogen on the dopaminergic system. For example, we measured a behavioural endophenotype of schizophrenia, prepulse inhibition, in ovariectomised female rats treated with estrogen and its analogues [173]. We showed that 17*β*-estradiol, raloxifene, and tamoxifen prevented the disruption of prepulse inhibition induced by the dopamine D1/D2 receptor agonist, apomorphine [173]. In another animal behaviour relevant to schizophrenia, 17*β*-estradiol treatment in combination with chronic antipsychotic haloperidol reduced amphetamine-induced locomotor hyperactivity in ovariectomised amphetamine-sensitized female rats [174]. Interestingly, this effect of estradiol was not observed when paired with saline treatment, suggesting that estradiol exerts antipsychotic properties that further potentiate the functional efficacy of haloperidol. However, the lack of a haloperidol treatment-only group in this study makes it difficult to ascertain this facilitatory effect. In contrast to these studies in female rats, the treatment of male rats with 17*β*-estradiol following gonadectomy has not been shown to effect the mRNA expression of enzymes involved in dopamine synthesis and metabolism [175]. This is in contrast to the increased expression of these enzymes seen in response to testosterone, suggesting that estrogen's effect on dopamine signalling may be sex-specific [175]. Overall, these studies suggest a protective action of estrogen, particularly in females, on the dopaminergic system.

#### 3.6.2. Estrogen Interaction with Serotonin

The advent of clinically effective atypical antipsychotics which have a higher affinity for serotonin receptors compared to typical antipsychotics, has highlighted a role for the serotonergic system in schizophrenia [160, 176, 177]. Further, postmortem studies have reported altered levels of several serotonin receptors in cortical and subcortical regions of the CNS in people with schizophrenia [178–182]. Sex differences in the regulation of serotonin signalling have been reported by some, where serotonin 5-HT1A receptor mRNA levels were lower in the amygdala and hypothalamus of female rats compared to males, whilst 5-HT2A receptor binding was higher in the female hippocampus [183]. Treating female rats with 17*β*-estradiol has been found to improve spatial working memory and increase the levels of serotonin in the prefrontal cortex [184]. PET studies of postmenopausal women showed that estradiol replacement therapy increased serotonin 5-HT2A receptor levels in the prefrontal cortex [185, 186]. However, serotonin 5-HT1A receptor levels were not altered following estradiol replacement therapy [187], suggesting estradiol's selective action on serotonin receptor subtypes. Another study reported a trend toward elevated plasma serotonin levels in postmenopausal women following estradiol replacement therapy, although this effect failed to reach statistical significance [188]. Furthermore, clinical studies in postmenopausal women showed that removing tryptophan (a biochemical precursor for serotonin synthesis) from the diet prior to, but not after, estradiol treatment can reduce dorsolateral prefrontal and cingulate cortex activation during working memory tasks [189]. Interestingly, a PET study conducted in healthy men found a positive correlation between the levels of the serotonin 5-HT2A receptor ligand [^18^F]altanserin and plasma levels of estradiol [190].

The effects of estradiol on serotonin receptor signalling may result from its capacity to modulate serotonin biosynthesis. In vitro studies in raphe cells have shown that ER*β* acts as a transcription factor for the tryptophan hydroxylase-2 gene, which encodes the enzyme involved in biosynthesis of serotonin from tryptophan [191]. Interestingly, it has been suggested that changes in serotonin biosynthesis in response to estradiol may vary following a prolonged disruption of estradiol signalling. Studies in macaques ovariectomised for 3 years report reductions in neurons positive for serotonin 5-HT1A receptor, serotonin transporter, and tryptophan hydroxylase-2 gene expression [192]. These changes were largely absent in macaques 5 months after ovariectomy, while another study reported increased numbers of serotonin positive neurons in macaques 4–7 months after ovariectomy [170], suggesting reduced serotonergic signalling may become more pronounced with chronic loss of estradiol [193]. Using the behavioural paradigm prepulse inhibition, we found that treatment of female ovariectomised rats with 17*β*-estradiol prevented a serotonin 5-HT1A receptor agonist-induced disruption of prepulse inhibition [21, 173, 194]. Similarly, we found that in healthy women pretreatment with estradiol prevented the disruption of prepulse inhibition induced by a partial serotonin 5-HT1A receptor agonist, buspirone [195]. Further, in these same women, we found that estradiol pretreatment prevented a further buspirone-induced potentiation in loudness dependence of the auditory evoked potential (LDAEP) [196]. This is a measure of early sensory processing that is thought to be primarily mediated by central serotonin function (reviewed in [197]). A high LDAEP, which is indicative of low serotonin neurotransmission, has been found to be tightly associated with the negative symptoms of schizophrenia [198]. These results are therefore suggestive of a protective role of estradiol against the sensory processing or gating deficits typically observed in schizophrenia patients. Overall, the above studies collectively substantiate the idea of estradiol interacting with multiple facets of the serotonergic system, through which it might exert protective actions against the cognitive, positive, and negative symptom domains of schizophrenia.

#### 3.6.3. Estrogen Interaction with Glutamate

Glutamate signals through two classes of receptors: the ionotropic receptors, which include the NMDA receptor, kainate receptor, and AMPA receptor subtypes; the metabotropic receptors, which include mGluR1-mGluR8 subtypes. The psychomimetic actions of drugs such as ketamine and phencyclidine, which are antagonists of the NMDA receptor, implicate the glutamatergic system in the pathophysiology of schizophrenia [199, 200]. Thus, glutamate's role in schizophrenia has been proposed to involve NMDA receptor hypofunction [201]. Postmortem studies of brains from people with schizophrenia show regionally discrete increases and decreases in both the levels of NMDA receptor [202–204] and the levels of other ionotropic and metabotropic glutamate receptors [202, 203, 205, 206]. Furthermore, regionally specific differences in the expression of the subunit components of these receptors are also reported in people with schizophrenia, which could affect receptor activity [202, 207, 208]. Chronic treatment with 17*β*-estradiol has been shown to modulate glutamate NMDA and AMPA receptor density in the rat brain [209]. Further, studies showing treatment with 17*β*-estradiol or selective estrogen modulators can alter the NMDA receptor subunit levels in the rat hippocampus [209, 210] suggest that estrogen may have varying effects on different areas of the brain in schizophrenia. However, these findings are contrasted by studies in macaques, showing treating ovariectomised animals with 17*β*-estradiol does not alter hippocampal AMPA receptor or NMDA receptor subunit expression [211]. Studies examining estradiol's effects on neurodegeneration and damage in cortical and hippocampal neuron cultures have shown that estradiol can be neuroprotective against the effects of glutamate mediated neurotoxicity, further supporting a role for estradiol in the modulation of glutamate signalling [212, 213]. Endogenous estradiol has been shown to enhance basal glutamatergic transmission and facilitate synaptic plasticity in the mouse medial prefrontal cortex [214].

Our understanding of how estradiol regulates glutamate signalling in the CNS has been advanced by recent in vitro studies examining how the glutamate pathways are affected by selective estrogen receptor modulators. Tamoxifen, which has both agonist and antagonist properties on ERs in different tissues, has been shown to increase glutamate reuptake [215], suggesting that estradiol could affect the broad changes in ionotropic and metabotropic glutamate receptor activity in schizophrenia by regulating the availability of glutamate in the synaptic cleft. Excitatory amino acid transporters (EAAT) control the uptake of surplus glutamate from the synaptic cleft. In primary astrocyte cultures, tamoxifen and raloxifene are both able to upregulate the mRNA and protein expression of the astrocytic glutamate transporters, EAAT1 and EAAT2, via NFkB mediated pathways [216]. The increase in EAAT2 expression in response to raloxifene has been shown to correspond to an increase in glutamate uptake, suggesting that the increase in EAAT levels results in a functional increase in EAAT activity [217, 218]. This upregulation of glutamate transporter gene expression by tamoxifen and raloxifene is mediated via ER*α* and ER*β* receptors as well as GPER via extracellular signal-regulated kinases, the epidermal growth factor receptor, and cAMP response element-binding protein-mediated regulation of the NF-*κ*B pathway [217, 218]. Treating astrocytic cells with estradiol has also been shown to increase EAAT1 and EAAT2 mRNA and protein expression, an effect that is attenuated by the estrogen receptor antagonist, ICI. Therefore, the effects of tamoxifen and raloxifene on astrocytic glutamate uptake are likely to result from the activation of the estrogen receptors and thus are comparable to the actions of estradiol [219].

Animal behavioural studies suggest estradiol's effects on glutamatergic signalling may be involved in behaviours relevant to schizophrenia. Estradiol is protective against NMDA receptor antagonist-induced impairments in the novel object recognition task [220, 221], suggesting that estradiol has the potential to affect glutamatergic dysfunction in schizophrenia. Postweaning social isolation in rats has been shown to result in increased prepulse inhibition and startle response as well as cognitive rigidity, which is reflective of schizophrenia-like symptoms. In male rats, these symptoms are associated with a dysregulation of the serotonin and dopaminergic system in the CNS. By contrast, social isolation in female mice has been shown to downregulate the expression of the NR1 NMDA receptor subunit and the GluR1 AMPA receptor subunit and PSD95 as well as synapsin, which is involved in glutamate release [222]. Whilst such studies suggest that estradiol plays a role in glutamatergic dysfunction in schizophrenia, estradiol treatment has not been shown to reduce deficits in prepulse inhibition in rats, caused by the NMDA receptor antagonist, MK-801 [21]. Thus, further evidence is needed to support a therapeutic effect of estradiol on glutamate dysfunction in schizophrenia. Overall, whilst substantial evidence supports the role of estradiol in modulating the glutamatergic system at the molecular level, how these mechanisms make an impact at the phenotypic level remains elusive and thus requires more in-depth investigation.

## 4. Summary and Conclusions

In summary, schizophrenia is a neuropsychiatric disorder that has shown robust gender differences in numerous aspects of the illness, including an earlier age of onset, a more severe course of illness, poorer antipsychotic treatment response, and adaptability to illness in male patients with schizophrenia compared to that of women. This review has highlighted the research that has been invested to understand the potentially protective effects of estradiol with respect to these gender differences in schizophrenia. The extent of this research ranges from molecular investigations that have clearly evidenced estradiol's intricate interactions with the major neurotransmitter systems in the brain, and especially those implicated in schizophrenia, to preclinical models of the illness that have shown estradiol's potential in either enhancing cognition and memory or reversing deficits that are reflective of the positive, negative, and cognitive symptoms of schizophrenia. Recent clinical trials have provided a promising outlook on the use of estradiol and the more recent use of selective estradiol receptor modulators, as an adjunctive treatment to antipsychotics for schizophrenia patients of both genders. Future studies investigating the mechanism underlying estradiol's protective action in schizophrenia are warranted; such research is also necessary in other psychiatric disorders where gender differences are observed, including depression and anxiety.
